# The benefits of hydrotherapy to patients with spinal cord injuries

**DOI:** 10.4102/ajod.v7i0.450

**Published:** 2018-05-16

**Authors:** Terry J. Ellapen, Henriëtte V. Hammill, Mariëtte Swanepoel, Gert L. Strydom

**Affiliations:** 1 School of Biokinetics Recreation and Sport, Physical Activity Sport and Recreation (PhASRec), North-West University, South Africa

## Abstract

**Background:**

Many patients with spinal cord injury (PWSCI) lead sedentary lifestyles, experiencing poor quality of life and medical challenges. PWSCI don’t like to participate in land-based-exercises because it’s tedious to perform the same exercises, decreasing their rehabilitative compliance and negatively impacting their well-being. An alternative exercise environment and exercises may alleviate boredom, enhancing compliance.

**Objectives:**

Discuss the benefits of hydrotherapy to PWSCI concerning underwater gait-kinematics, thermoregulatory and cardiovascular responses and spasticity.

**Methodology:**

A literature surveillance was conducted between 1998 and 2017, through the Crossref meta-database and Google Scholar, according to the PRISMA procedures. Key search words were water-therapy, aquatic-therapy, hydrotherapy, spinal cord injury, rehabilitation, human, kinematics, underwater gait, cardiorespiratory, thermoregulation and spasticity. The quality of each paper was evaluated using a modified Downs and Black Appraisal Scale. The participants were records pertaining to PWSCI and hydrotherapy. The outcomes of interest were: hydrotherapy interventions, the impact of hydrotherapy on gait-kinematics, thermoregulation during water submersion and cardiorespiratory function of PWSCI. Omitted records included: non-English publications from before 1998 or unrelated to hydrotherapy and PWSCI. The record screening admissibility was performed as follows: the title screen, the abstract screen and the full text screen.

**Results:**

Literature search identified 1080 records. Upon application of the exclusion criteria, 92 titles, 29 abstracts and 17 full text records were eligible. Only 15 records were selected to be included in this clinical commentary. Evidence shows a paucity of randomised control trials (RCT) conducted in this field.

**Conclusion:**

Hydrotherapy improves PWSCI underwater gait-kinematics, cardiorespiratory and thermoregulatory responses and reduces spasticity.

## Introduction

Hydrotherapy, also known as aquatic or water therapy, has long been perceived as an effective, yet underutilised, therapeutic modality (Kesiktas et al. 2004). The benefits of hydrotherapy include enhanced aerobic capacity, improved muscle strength and endurance, increased joint range of motion (anti-spasticity), as well as decreased muscle fatigue and joint pain, enhanced cardiorespiratory functioning and a reduced cardiometabolic risk profile (Kesiktas et al. 2004). The majority of patients with spinal cord injury (PWSCI) lead sedentary lives, associated with a poor cardiometabolic profile (diabetes mellitus, increased insulin resistance, decreased insulin sensitivity, increased adiposity, obesity and body mass index as well as poor cardiorespiratory function) (Nooijen et al. 2016). Attempts to combat the poor cardiometabolic risk profile of PWSCI usually involve upper limb land-based exercises (Tweedy et al. 2016). La Fountaine et al. (2015) reported that these upper limb exercises are not as effective as lower limb exercises with regard to expending energy. A primary goal of exercising is to increase PWSCI’s energy expenditure, thereby improving their poor cardiometabolic risk profile (La Fountaine et al. 2015). In order to increase energy expenditure, the rehabilitation programme’s frequency, intensity and duration are increased, so that in turn it often leads to more upper limb injuries (Ellapen et al. 2017). Ellapen et al. (2017) reported that habitual use of the same upper body exercises leads to overuse orthopaedic injuries and boredom, resulting in poor rehabilitative exercise adherence. Strydom et al. (2009) reported that variation in habitual exercise and rehabilitative regimes increases patient adherence and subsequently is able to positively impact the realisation of the programme objectives. Hydrotherapy provides the following alternative options to land-based exercises: (1) a different rehabilitative environment, (2) the prescription of different upper limb and core exercises and (3) the opportunities for group and/or individual rehabilitation sessions with the exercise therapist (thereby increasing social interaction) (Kesiktas et al. 2004).

The therapeutic benefits of hydrotherapy relate to the following fundamental principles of hydrodynamics: (1) density, (2) drag, (3) buoyancy, (4) hydrostatic pressure and (5) thermodynamics. Density is explained using Archimedes’ law of buoyancy, that the upward buoyant force exerted on an object immersed in water is equal to the weight of the water (or fluid) that the object displaces. This means that the human body, being of lower density than water, is subjected to a buoyant force (bringing the body to the surface) equal to the weight of the water that is displaced by the body’s immersion (Becker 2009).

Thus, buoyancy occurs when a person is immersed in water, producing water displacement and progressively offloading the force of gravity on the immersed joints. By immersing the patient in water up to the cervical, thoracic (xiphoid process) and hip (pubic symphysis) joints, the therapist is able to offload 85%, 60% and 40%, respectively, of the patient’s individual body weight (gravity) that would normally weigh down on the submersed joints (Becker 2009). Buoyancy has great therapeutic value by allowing PWSCI to become mobile in the water without the resistance of gravity. The water becomes a dynamic fluid medium that allows PWSCI to safely, spontaneously and independently exercise and stabilises their lumbopelvic hip, thoracic and cervical muscles without relying on the use of their upper limbs in order to support their posture during the exercise, as is often the case during land-based exercises. This can be the key in the prevention of upper limb overuse injuries.

Drag force refers to the size of the internal resistive friction against movement in the fluid medium (water) (Poyhonen et al. 2000). The magnitude of drag increases as more force is exerted by the person, but is immediately neutralised (returning to zero) upon the cessation of movement, thereby providing accommodative hydro-resistance and thus preventing injuries in a similar manner to land-based isokinetic accommodative resistance (Poyhonen et al. 2000).

Hydrostatic pressure is the pressure exerted by the water during equilibrium at a given point during submersion, caused by gravity. Hydrostatic pressure is directly influenced by the density of water and by the depth of submersion. Hydrostatic pressure assists in the dissipation of oedema, in the gradual increase in joint range of motion and in combatting spasticity (Becker 2009).

Thermodynamics refers to water’s ability to transfer heat. A significant therapeutic value of hydrotherapy depends on its ability to retain heat, as well as the transfer thereof. Fortunately, water is an efficient conductor, transferring heat 25 times faster than that of an equivalent volume of air (Bailey et al. 2007). Hydrotherapy can be used at a variety of temperatures: Ice water baths are often used post-training by athletes to reduce the effect of delayed-onset muscle soreness, to promote the dissipation of inflammation and to quicken their recovery from training (Bailey et al. 2007). Warm water immersion decreases muscle pain, increases vasodilation and blood circulation, lowers heart rate and enhances thermoregulatory responses (Munguia-Izquierdo & Legaz_Arrese 2007; Ingram et al. 2009). The temperatures of typical hydrotherapy pools range from 33.5 °C to 35.5 °C (Bailey et al. 2007). Heat transmission starts immediately upon initial water submersion primarily because the human body has a lower heat capacity than water (Bailey et al. 2007).

Over and above the benefits regarding heat conduction, water has further benefits related to respiration: *Boyle’s law* suggests that the volume of any gas varies inversely with the pressure exerted upon it. Greater submersion depth, therefore, increases the hydrostatic pressure against the thoracic cage, thereby inversely impacting its lung volume. The therapeutic benefit is the increased respiratory cost during water submersion, which expends more calories and improves respiratory efficiency, positively impacting one’s cardiometabolic profile (Becker 2009).

According to the authors’ knowledge, there have been two reviews published on aquatic therapy in relation to PWSCI (Li, Khoo & Adan 2017; Recio, Stiens & Kubrova 2017). Li et al. (2017) is the only systematic review that evaluates the quality of the research of aquatic therapy and exercise prescribed to PWSCI. However, Li et al. (2017) did not discuss the rehabilitation and exercise physiology mechanisms, but highlights the value of hydrotherapy. Recio et al. (2017) only describe the clinical anti-spasticity and ventilatory benefits of hydrotherapy for PWSCI; they neither describe the methodology used to find the papers nor the PWSCI’s underwater gait kinematics, thermoregulatory and cardiorespiratory responses to aquatic therapy. This commentary combines the elements of rigorous methodology undertaken by Li et al. (2017) in their systematic review and the discussion of the therapeutic benefits of hydrotherapy for PWSCI. Further, the novelty of this commentary lies in the biomechanical discussion of hydrotherapy, specifically considering PWSCI’s gait kinematics, cardiorespiratory, spasticity and thermoregulatory responses. The aim of this commentary is to determine the effect of hydrotherapy on PWSCI’s gait kinematics, muscle spasticity, cardiorespiratory and thermoregulatory responses.

## Methods

The authors followed the standard practices for systematic reviews: Preferred Reporting Items for Systematic Reviews and Meta-Analyses (PRISMA).

**FIGURE 1:**
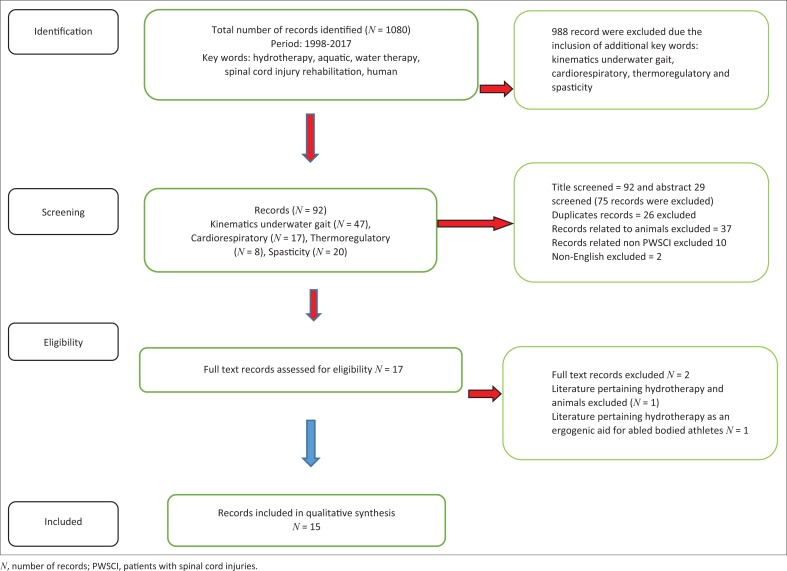
Flow chart of the review process.

**Information sources and searches:** A literature search of peer-reviewed records was conducted using the following search engine: Crossref meta-database, which is an academic database comprising of the following search engines: PubMed, Medline, Science Direct, Ebscohost, CINAHL and Google Scholar (Figure 1). The keywords used in the literature search were water therapy, aquatic therapy, hydrotherapy, spinal cord injury, rehabilitation, human, kinematics, underwater gait, cardiorespiratory, thermoregulation and spasticity. The screening eligibility of records was performed in the following three steps: (1) the title screen, (2) the abstract screen and (3) the full text screen. The records were screened by TJE, HVH, MS and GLS.

**Eligibility criteria:** The participants in this study were records pertaining to PWSCI and hydrotherapy; the intervention was not necessarily a therapeutic intervention but is interpreted as an exposure, namely the effect of hydrotherapy on the well-being of PWSCI. The outcomes of interest were (1) hydrotherapy interventions for PWSCI, (2) the impact of hydrotherapy on PWSCI gait kinematics, (3) the effect of hydrotherapy on PWSCI thermoregulation during water submersion and (4) the impact of hydrotherapy on PWSCI cardiorespiratory function. The exclusion criteria were (1) publications prior to 1998, (2) literature pertaining to hydrotherapy and animals, (3) literature related to hydrotherapy as an ergogenic aid among able-bodied athletes, (4) the impact of hydrotherapy on the health and well-being of able-bodied athletes and (5) non-English papers.

### Study selection: The appraisal of the quality of records

All records were filtered based on the appropriateness of their title and the inclusion criteria. The quality of each record was appraised using a modified Downs and Black Appraisal Scale, which examines the quality of randomised controlled trials and non-randomised papers (Downs & Black 1998) (Table 1). The evaluation of the quality of each record reduced the risk of researcher biasness. The modified Downs and Black checklist was adopted as not all the items on the original checklist were related to this paper, as similarly cited in Ellapen et al. (2017). The modified checklist comprises 16 questions with a maximum of 16 points. Answers were given a score of either 0 (no) or 1 (yes). The questions adopted from the Downs and Black Appraisal Scale were questions number 1, 3, 4, 5, 6, 10, 11, 12, 13, 14, 18, 20, 21, 22, 23 and 27. These questions are categorised into four sections in order to assess the overall quality of each paper (Table 2). The sections include reporting prowess (*n* = 5 questions), external validity (*n* = 3 questions), internal validity (*n* = 3 questions) and power of significance (*n* = 5 questions) (Downs & Blacks 1998). All authors were allowed to dispute the scoring of each record. The authors would then discuss scores and adopted the mutually accepted score. The sum of these scores was then converted to a percentage so as to rate the overall quality of the individual papers (Downs & Black 1998). The overall quality of the papers was graded using a scale defined as follows: < 50% (weak), 50% – 69% (fair), 70% – 79% (good) and < 80% (very good) (Downs & Black 1998).

**TABLE 1:** Appraisal of records according to the modified Downs and Black Appraisal Scale.

Authors	Downs and Black Appraisal					

	Reporting (*n* = 5)	External validity (*n* = 3)	Internal validity (*n* = 3)	Power (*n* = 5)	Total (*n* = 16)	Grading % = *x*/16 x 100
Zamparo and Pagliaro (1998)	5	2	2	1	10	62.5% (fair)
Gass and Gass (2001)	5	2	2	3	12	75% (good)
Gass, Gass and Pitetti (2002)	5	2	2	1	10	62.5% (fair)
Kesiktas et al. (2004)	5	2	2	3	12	75% (good)
Prosser (2007)	4	3	1	1	9	56.2% (fair)
Becker (2009)	4	1	1	1	7	43.7% (weak)
Lucksch et al. (2013)	5	3	2	3	13	81.25% (good)
Tamburella et al. (2013)	5	2	2	3	12	75.0% (good)
Jung et al. (2014)	5	3	3	5	16	100.0% (very good)
Stevens et al. (2014)	5	2	2	1	10	62.5% (fair)
Stevens and Morgan (2015)	5	2	2	1	10	62.5% (fair)
Tweedy et al. (2016)	4	1	1	1	7	43.7% (weak)
Li et al. (2017)	4	0	1	1	6	37.5% (weak)
Recio et al. (2017)	4	0	1	1	6	37.5% (weak)
Wall, Falvo and Kesten (2017)	5	3	0	1	9	56.2% (fair)
Overall rating	4.6	1.3	1.6	1.8	9.9	62.0% (fair)

*N*, number; x, sum of Downs and black appraisal.

**TABLE 2:** Chronological overview of the characteristics and findings of the records (*n* = 15).

Authors	Characteristics of the study			

	Type of study	Sample	Method	Findings
Zamparo and Pagliaro (1998)	Experimental Non-randomised control (RCT)	23 spastic paresis patients (body mass: 80.2 kg ± 13.2 kg, age: 56.0 ± 14.6 years, number of years since spasticity: 10.7 ± 6.6 years) underwent a hydrotherapy intervention for 45 min/daily for 14 days. The cohort comprised of 12 affected by hemiparesis, 4 by multiple sclerosis and 7 incomplete SCI. The hydrotherapy included passive and active movements in 32°C seawater, free swimming, and water-immersion walking.	Energy expenditure and kinematic gait characteristics were measured pre- and post-hydrotherapeutic intervention using the overall steady-state oxygen consumption over a variety of walking speeds as a measure of energy expenditure.	The hydrotherapeutic intervention successfully lowered subjects’ energy expenditure and improved kinematic gait characteristics especially at slow speeds.
Gass and Gass (2001)	Experimental Non-RCT	Paraplegic group: 5 males, age: 37 ± 4 years, body mass: 62.8 kg ± 4 kg Able-bodied group: 6 males, age: 22 ± 1 year, body mass: 81.0 kg ± 3.0 kg	Pre-test and post-test measurements included VO_2_max, VCO_2_max, VEmax, heart rate (HR), oesophageal temperature (Tes) and sweat rate. Venous blood was analysed before and during water immersion to estimate plasma volume. Subjects sat in nipple height water at 39°C for 60 min while propelling a wheelchair on a treadmill (paraplegics) or cycle ergometer (able-bodied).	Repeated warm water immersion for prolonged periods does produce significant thermoregulatory adaptations in paraplegic individuals, but not to the same degree as in able-bodied individuals.
Gass et al. (2002)	Experimental Non-RCT	Four physically conditioned tetraplegic males participated in 3 experiments. Age: 38 ± 4.5 years, body mass: 68.6 kg ± 11.9 kg, number of years since injury: 15.5 ± 3.6 years	The first (pre-test) experiment measured the participants’ VO_2_max through an incremental test to exhaustion on their wheelchair on a treadmill. The second experiment involved them wheeling their wheelchairs on a treadmill at 65% of their VO_2_max for 40 min. The third experiment involved them wheeling their wheelchairs at 65% of their VO_2_max for 60 min immersed in 39°C water. For each experiment venous blood was analysed pre-, during and post-experiment. Furthermore, haemoglobin, haematocrit, fluctuations of plasma volume, heart rate, rectal temperature and sweat rate were noted. The washout period between experiments was 1 week.	Hydrotherapy exercising produced a lower heart rate, plasma noradrenalin concentration, and increase plasma volume, as compared to land-based exercising.
Kesiktas et al. (2004)	Experimental Non-RCT	Experimental or hydrotherapy group: 10, mean age: 32.1 years, gender: 8 males and 2 females, tetraplegics: 3, paraplegics: 7, number of years injured: 7.7 years Control group: 10, mean age: 33.1 years, gender: 7 males and 3 females, tetraplegics: 3, paraplegics: 7, number of years injured: 8.6 years	The experimental group underwent 20 min of hydrotherapy (3 sessions/week) for 10 weeks, water temperature: 71°F (21.6°C). This accompanied their conventional rehabilitation (passive ROM, oral Baclofen and psychotherapy). The control group underwent their conventional rehabilitation.	The experimental group experienced greater reduction in spasticity and a reduced ingestion of Baclofen as compared to the control group (*p* < 0.01).
Prosser (2007)	Case study	A 5-year-old girl sustained a C4 lesion. Number of months since injury: 5	Underwent 5 months of physical therapy (7 days/week) in addition to 2–4 hydrotherapy sessions per week. Hydrotherapy focused on upright mobility, lower limb weight bearing and walking.	After 5 months of intensive physical therapy involving hydrotherapy the patient was able to walk.
Becker (2009)	Review	Records pertaining to the effect of hydrotherapy as a rehabilitation modality.	Does not describe the methods adopted.	Hydrotherapy is an effective therapeutic modality.
Lucksch et al. (2013)	Experimental RCT	Experimental group consisted of 9 male PWSCI, whose mean age (39 ± 14.2 years), stature (170 cm ± 7 cm) and body mass (67 kg ± 9.5 kg). Control group consisted of 10 healthy males, whose mean age (24.4 ± 3.5 years), 171 cm ± 4 cm and body mass (66 kg ± 4.1 kg).	Participants walked at self-selected speed in a heated pool at xiphoid process level. PWSCI were allowed to hold the researcher’s hand. The body segments and joint angles coordinates in the sagittal plane were measured with SIMI motion software.	Underwater gait kinematics of the PWSCI improved in regards to decreased knee flexion in their stance phase.
Tamburella et al. (2013)	Experimental Non-RCT	15 incomplete spinal cord injured patients and 15 able-bodied persons in the control group.	The land-based and water-immersion gaits of both groups were analysed with regard to the following kinematic variables: speed, stride length and stance phase.	Hydrotherapy improved the gait of PWSCI similar to the kinematic characteristics as of the control, with regard to speed, stride length and stance phase.
Jung et al. (2014)	Experimental RCT	Aquatic group: 10 (7 males and 3 females), age: 42.1 ± 10.6 years, height: 1.7 m ± 0.1 m, body mass: 64.2 kg ± 11.2 kg, time since injury: 8.5 ± 3.9 years. Land group: 10 (5 males and 5 females), age: 51.1 ± 8.7 years, body mass: 1.63 m ± 0.9 m, time since injury: 8.4 ± 3.4 years.	Both groups trained for 60 min, three sessions per week for 8 weeks. Pre-test includes forced vital capacity (FVC), forced expiratory flow rate (FER) forced expiratory volume in 1 s (FEV_1_), FEV_1_/FVC.	The aquatic group significantly enhanced their FVC, FER, FEV1 and FEV1/FVC (*p* < 0.05). The land group only improved their FER (*p* < 0.05).
Stevens et al. (2014)	Experimental Non-RCT	11 adults with incomplete-SCI underwent 3 sessions per week for 8 weeks of underwater treadmill training.	The experimental parameters were lower-extremity strength, balance, preferred and rapid walking speeds, 6-min walk distance and daily step activity.	Patients significantly enhanced their lower extremity strength, balance walking speeds, 6 min walking distance and daily step activity.
Stevens and Morgan (2015)	Experimental Non-RCT	7 males and 4 females with incomplete-SCI, underwent 3 sessions of underwater treadmill walking weekly for 8 weeks. Age: 48 ± 13 years, number of years since injury: 5 ± 8 years.	The experimental parameter was heart rate during the last 15 s of the walking.	Habitual underwater treadmill walking successfully reduced the heart rate among patients with incomplete-SCI.
Tweedy et al. (2016)	Review	A review of the common rehabilitation exercises applicable to PWSCI.	Does not describe the methods adopted.	This consensus statement paper provides guidelines for exercise rehabilitation of PWSCI, including hydrotherapy. However, the frequency, duration and intensity are omitted.
Li et al. (2017)	Review	A systematic review of the efficacy of hydrotherapy as a therapeutic modality for PWSCI.	The following databases were searched: MEDLINE, CINAHL, EMBASE, PsychInfo, SPORTDiscus and Cochrane Center Register of Controlled Trials. 8/276 studies met the inclusion criteria, of which none showed high research quality. 4 studies assessed physical function outcomes and 4 studies evaluated aerobic fitness. Significant improvements on these 2 outcomes were generally found. Body composition, muscular strength and balance were rarely reported.	There is weak evidence supporting aquatic exercise training as improving physical function and aerobic fitness among adults with spinal cord injury.
Recio et al. (2017)	Review	Records pertaining to the effect of hydrotherapy as a rehabilitation modality.	Does not describe the methods adopted.	Aquatic therapy has been demonstrated to be a valuable therapeutic modality for the management of PWSCI; however, it is underutilised.
Wall et al. (2017)	Case study	An incomplete PWSCI underwent two sessions of hydrotherapy per week for 6 weeks. Two post follow-up evaluations were conducted after the hydrotherapeutic intervention.	The post therapeutic evaluations identified significant changes in the Walking for Spinal Cord Injury Index II, Spinal Cord Injury Ambulation Index gait parameters and gait speed.	The participants showed significantly improved clinical gait kinematics after hydrotherapy.

PWSCI, patients with spinal cord injury; SCI, spinal cord injury.

## Results

The literature review identified 1080 records by the use of the key search words (water therapy, aquatic therapy, hydrotherapy, spinal cord injury, rehabilitation and human). The application of additional key words (kinematics underwater gait, cardiorespiratory, thermoregulation and spasticity) resulted in 92 records. All the titles of each record were screened (*n* = 92); however, only 29 abstracts were screened. Thirty-seven animal records, 26 duplicate records, 10 records pertaining to non-PWSCI and 2 non-English records were excluded. The remaining 17 full text records were reviewed. Two full text records comprised of one animal record and the other that pertained to the adoption of hydrotherapy as an ergogenic aid. The remaining 15 records comprised of 4 systematic reviews pertaining to PWSCI (but 2 specific to hydrotherapy), 7 non-randomised control trials, 2 randomised control and 2 case studies (Table 2). Table 1 assesses the quality of each record according to the modified Downs and Black Appraisal Scale (in an attempt to eliminate risk of biasness). A descriptive overview of the characteristics and findings of the studies is found in Table 2. A total of 142 participants were reported (but 83 PWSCI), with sample sizes varying from 1 to 30 and participant age varying from 5 to 70 years. Five studies provided kinanthropometric characteristics, whereas 5 studies considered the number of years injured, and 10 studies described the aquatic exercise intervention. The overall quality of the studies was rated as fair (62.0%) (Table 1).

## Discussion

The discussion will focus on the empirical findings of the impact of hydrotherapy on the gait kinematics of PWSCI as well as their thermoregulatory, spasticity and cardiorespiratory responses.

### Kinematic gait analyses

The kinematic gait analysis studies involved the review of the form or technique of PWSCI underwater walking. Zamparo and Pagliaro (1998), Prosser (2007) and Tamburella et al. (2013) all concur that the patients’ gait kinematics, walking speed and stride length improved after the completion of hydrotherapy. However, the aforementioned authors failed to describe the biomechanical mechanism facilitating PWSCI-enhanced gait kinematics. It was postulated that hydrostatic pressure combined with the effects of buoyancy enhanced the patients’ lumbopelvic hip complex form and force closure, thereby enhancing their underwater gait kinematics. Buoyancy helped elevate the contralateral hip during the stance phase, thereby decreasing the muscle contraction force required to elevate the contralateral hip. Furthermore, buoyancy negated the effects of gravity, enhancing the swing phase of the ipsilateral hip. Zamparo and Pagliaro (1998) also reported that PWSCI energy expenditure was lower during underwater walking as compared to land-based walking at specific speeds, which allowed them to walk for longer. However, Zamparo and Pagliaro’s (1998) study was limited by their research design (experimental, non-RCT and without concurrent controls). Therefore, Zamparo and Pagliaro (1998) recommended future empirical investigations adopting experimental RCT procedures with comparative concurrent controls in order to validate the findings of the prospective studies. However, Lucksch et al. (2013) and Jung et al. (2014) heeded to Zamparo and Pagliaro’s (1998) recommendations. This computes into two RCT out of 92 records (2.1%) published during the period of 1998 to 2017 pertaining to human PWSCI and hydrotherapy. The authors of this article strongly encourage more RCT examining the effects of hydrotherapy on PWSCI needs to be completed and published. This empirical evidence will help to encourage exercise therapists to prescribe hydrotherapy as a supplement to other management practices of PWSCI. Gass and Gass (2001) and Gass et al. (2002) confirmed Zamparo and Pagliaro’s (1998) postulation that increased exercise duration will augment energy expenditure of paraplegic patients in a manner that will positively impact their cardiometabolic profile. Tweedy et al. (2016) and Ellapen et al. (2017) both concur that lower limb exercises (i.e. walking and strengthening) expend more energy than upper limb exercises. Therefore, lower limb exercises are needed to increase PWSCI energy expenditure to help improve their cardiometabolic risk profile. The authors of this study recommend that PWSCI should engage in hydrotherapeutic walking before land walking. The hydrotherapeutic walking will serve to condition the PWSCI lower limb neuromuscular system, preparing them for land walking and simultaneously increasing energy expenditure, lowering their cardiometabolic risk profile.

### Thermoregulatory response to exercising in warm water submersion

Exercising while submerged in warm water lowers the heart rate and enhances thermoregulatory responses, thereby prolonging the PWSCI’s ability to exercise and thus increasing their aerobic capacity (Gass & Gass 2001; Gass et al. 2002). The prolonged exercising during water submersion increases the patient’s energy expenditure, thereby lowering their cardiometabolic risk profile. Water is an excellent conductor of heat, which enhances patients’ ability to effectively thermoregulate their bodies when exercising and maintains a low core temperature (Becker 2009). This further physiological adaptation also contributes to the ability of patients to exercise for longer, thereby improving their cardiorespiratory function and energy expenditure (Gass et al. 2002; Becker 2009).

### Decreased spasticity response to hydrotherapy

Kesiktas et al. (2004) reported that PWSCI experienced a significant reduction in muscle spasticity with a reduced dosage of oral baclofen because of hydrotherapy. This is the only study, among numerous clinical reports of reduced muscle soreness, spasticity and increased joint range of motion among arthritic patients, who also experience spasticity (Eversden et al. 2007), to report this result. Becker (2009) reported that the physiological rationale behind the efficacy of hydrotherapy on spasticity, muscle soreness and joint range of motion is enigmatic. Therefore, further clinical investigation should be undertaken so as to unravel the physiological mechanism of the efficacy of hydrotherapy in relation to the aforementioned maladies.

### Cardiorespiratory benefits

The patient’s cardiorespiratory adaptations are based on Boyle’s law. When a person is submersed in water, the hydrostatic pressure against the body increases, thereby decreasing their lung volume (Becker 2009). Greater submersion depth increases the hydrostatic pressure, making breathing more costly. Becker (2009) reported that the patient’s vital capacity is reduced by 6% – 9% because of compression by external hydrostatic pressure which counteracts inspiratory muscle action. Energy expenditure at rest increases by 60% during neck-level submersion which in turn enhances inspiratory muscle strength and endurance, serving as an effective respiratory rehabilitative exercise medium able to counteract respiratory diseases (Taylor & Morrison 1999; Becker 2009). Pachalski and Mekraski (1980) reported that PWSCI gained a greater cardiorespiratory fitness improvement by following an aquatic exercise programme as compared to land-based exercises. Van Houtte, Vanlandewijck and Gosselink (2006) and Jung et al. (2014) reported that respiratory muscle rehabilitation conditioning programmes increase the expiratory muscle strength, vital capacity and residual volumes of PWSCI.

When a person is submerged in water, blood is displaced towards the heart, thereby enhancing central venous return, which in turn increases arterial and ventricular filling and results in a subsequent decrease in heart rate (Becker 2009). There is a significant increase in end-diastolic volume, producing a larger stroke volume. During aquatic exercising, maximal oxygen consumption is greater than that of land-based exercise, allowing for greater energy expenditure at slower speeds and prolonged activity (Becker 2009). During neck-level submersion, there is a decrease in sympathetic nervous activity which reduces peripheral resistances, thereby allowing greater venous return (Becker 2009). Stevens and Morgan (2015) reported that habitual underwater treadmill walking reduces PWSCI heart rate, suggesting enhanced cardiorespiratory function. It is hypothesised that the lower exercise heart rate experienced during underwater walking combined with effective thermoregulatory response will increase exercise duration or walking distance, thereby increasing energy expenditure, which in turn will positively impact on the cardiometabolic profile of PWSCI. It should, however, be noted that these suppositions require clinical validation.

## Conclusion

Hydrotherapy aids in reducing PWSCI muscle spasticity and cardiometabolic risk profiles, while favourably enhancing underwater gait kinematics and cardiorespiratory capacity. However, more RCT should be undertaken to increase the present body of knowledge.
